# Advances in noninvasive myelin imaging

**DOI:** 10.1002/dneu.22552

**Published:** 2017-11-10

**Authors:** Florence Heath, Samuel A. Hurley, Heidi Johansen‐Berg, Cassandra Sampaio‐Baptista

**Affiliations:** ^1^ Oxford Centre for Functional MRI of the Brain, Nuffield Department of Clinical Neurosciences John Radcliffe Hospital, University of Oxford Oxford OX3 9DU United Kingdom; ^2^ Departments of Neuroscience and Radiology, 1111 Highland Ave University of Wisconsin – Madison Madison Wisconsin 53705

**Keywords:** Myelin, MRI, magnetization transfer, myelin water imaging

## Abstract

Myelin is important for the normal development and healthy function of the nervous system. Recent developments in MRI acquisition and tissue modeling aim to provide a better characterization and more specific markers for myelin. This allows for specific monitoring of myelination longitudinally and noninvasively in the healthy brain as well as assessment of treatment and intervention efficacy. Here, we offer a nontechnical review of MRI techniques developed to specifically monitor myelin such as magnetization transfer (MT) and myelin water imaging (MWI). We further summarize recent studies that employ these methods to measure myelin in relation to development and aging, learning and experience, and neuropathology and psychiatric disorders. © 2017 The Authors. Developmental Neurobiology Published by Wiley Periodicals, Inc. Develop Neurobiol 78: 136–151, 2018

## INTRODUCTION

Myelin is a sheath of multi‐layered specialized membrane that is formed around axons in the central nervous system (CNS) by oligodendrocytes. It is composed primarily of lipids, around 70%, along with a large variety of proteins (Jahn et al., 2009). Myelin internodes are separated by nodes of Ranvier, the short 1μm lengths of axon through which action potentials are transmitted. As a consequence, the speed of action potential propagation during synaptic transmission is significantly increased and neuronal energetic costs reduced. Oligodendrocytes differentiate from oligodendrocyte precursor cells (OPCs), which play a vital role during brain development but also in the adult CNS (Young et al., 2013). There is an intimate relationship between oligodendrocytes and the neurons they myelinate (Fields, 2015). For instance, electrically active neurons have been shown to regulate myelination (Wake et al., 2011).

Myelin development occurs well into adulthood (Benes et al., 1994). Imaging studies in humans demonstrate that white matter continues to develop throughout the life span until the early thirties and it starts to decline around 50 years of age (Sowell et al., 1999; Giedd, 2004; Lebel and Beaulieu, 2011; Sexton et al., 2014). Myelin is vital during normal brain development and has been linked with cognitive ability (Grydeland et al., 2013). Projection and commissural fibers are mostly developed by late adolescence, however association tracts are still under development during adulthood (Giedd, 2004; Lebel and Beaulieu, 2011). Myelin is necessary for healthy functioning of the CNS and demyelinating diseases such as multiple sclerosis have devastating, wide spread cognitive and motor symptoms (Murray, 2006). More recently, the role of myelin in experience dependent plasticity has started to be explored. Myelin plasticity could potentially be achieved by increased differentiation of OPCs, as well as changes in thickness, number and/or length of sheaths (Fields, 2015). Experiments have shown that experiences such as enriched environment and learning can induce such changes (Blumenfeld‐Katzir et al., 2011; Sampaio‐Baptista et al., 2013; Keiner et al., 2017) that can be indirectly detected with MRI in humans (Scholz et al., 2009; Sagi et al., 2012; Hofstetter et al., 2013).

To date, most neuroimaging studies in humans of white matter microstructure have used diffusion tensor imaging (DTI) to quantify tissue properties. For example, fractional anisotropy (FA), a commonly used DTI scalar parameter, quantifies the directional dependence of water diffusion, and has been shown to be sensitive to changes in myelin (Gulani et al., 2001). However, DTI does not detect changes in myelination alone and its measures can be affected by several features of the tissue microstructure, such as axon density, caliber, cell swelling, fiber architecture and myelin thickness (Beaulieu, 2009). Although DTI remains a useful and sensitive tool to assess brain microstructure, recent advances in MRI techniques open the possibility of development and routine use of methods that are more specific to myelin. MR based methods to estimate the myelin content in CNS tissues fall broadly into four categories: those based on the magnetization transfer (MT) effect, myelin water imaging (MWI), susceptibility mapping, and techniques mapping MR signal to patterns of cortical myelination. The aim of this review is to describe some of these techniques in simple technical terms to nonimaging experts. We further outline recent research using these methods to image myelin changes in the context of: human development and aging; skill learning, physical exercise and memory training; as well as neuropathology and psychiatric disorders.

## MR METHODS FOR MYELIN IMAGING

### MR Contrast: T1, T2, and Susceptibility

Most MR imaging is performed to measure signal from protons, typically from either bulk water or fat in the body. Tuned radiofrequency (RF) pulses are used to generate a signal from these protons (hence the “resonance” in MRI), combined with magnetic field gradients which are used to spatially encode these signals into images. By changing the timings of the application of RF and gradients, one sensitizes the MR signal to generate contrast from tissues due to differences in the microstructural and chemical environment of the measured water, similar to the application of stains to generate contrast to different properties of tissue microstructure in sectioned tissue samples (Alexander et al., 2011). In this section, we will specifically discuss sequences that sensitize the MR signal to various features of myelin (Table 1).

**Table 1:** List of MRI Terms and Parameters Relevant to Myelin Imaging

Abbreviation	Name	Description
f	Bound pool fraction	The ratio of bound protons to bound free protons, based on quantitative modeling of the magnetization transfer effect
MET2	Multi‐exponential T2	Techniques to fit MRI signal to multiple T2 compartments to attribute signal coming from multiple pools of water within CNS tissue
MPM	Multi‐parameter mapping	A technique to simultaneously map one or more relaxation parameters
MT	Magnetization transfer	An observed attenuation of observed MR signal in the presence of off‐resonance irradiation and macromolecules (proteins)
MTR	Magnetization transfer ratio	A parameter map showing the ratio of an image with MT weighting, divided by an image with no MT weighting
MWF	Myelin water fraction	The fraction of MR signal coming from water trapped within the lipid bilayers of water
MWI	Myelin water imaging	A family of MR techniques to quantify the fraction and relaxation parameters of water trapped within the lipid bilayers of water
PSR	Pool size ratio	The ratio of bound to free water pools, based on quantitative modeling of the magnetization transfer effect
QMT	Quantitative magnetization transfer	An imaging technique to quantify MT parameters (bound pool fraction, exchange rate, and relaxation parameters)
T1	Spin‐lattice relaxation	The rate at which excited protons give up their energy and return to the ground state.
T2	Spin‐spin relaxation	The rate at which MR signal decays away due to spins interacting with one another
T2*	Reversible spin‐spin relaxation	The rate at which MR signal decays away due to local variations in the magnetic field.
SWI	Susceptibility weighted imaging ‐	A gradient echo image with combined magnitude and signal phase information to highlight differences between susceptibility of tissues
QSM	Quantitative Susceptibility Mapping ‐	A technique to quantify the susceptibility shift of tissues using SWI

Contrast seen in magnitude MR images is driven by several intrinsic parameters of nuclear spins. Spin‐lattice relaxation time (T1) represents the time it takes for a perturbed system to return to equilibrium, or how quickly spins in a system interact with and give away energy to their surroundings. T1 becomes shorter as the water's environment contains more microstructural features, such as myelin and other macromolecules. Spin‐spin relaxation time (T2) describes how spins interact with each other, leading to dephasing and exponential decay of observed signal. T2 becomes shorter when spins are in a more geometrically restricted environment, a property that can be exploited to differentiate and assign the signal in MR to specific compartments within the CNS. Both T1 and T2 become longer with increasing water content in tissue.

In addition to intrinsic nuclear properties, magnetic susceptibility (henceforth referred to as susceptibility) is the degree to which a tissue or material is magnetized by an external magnetic field, and thus describes how the magnetic environment of MR is perturbed by the material placed within it (Deistung et al., 2017). Materials that are diamagnetic tend to become magnetized in a direction opposing an applied magnetic field, have a lower relative susceptibility value and reduced effective local magnetic field. Materials that are paramagnetic become magnetized parallel to an external magnetic field, have a higher relative susceptibility and increase the effective local magnetic field. Unlike T1 and T2, which locally affect the signal within each voxel, the influence of tissue susceptibility is nonlocal and affects the MR signal over a large portion of the imaged object. An example of this is the case of large susceptibility differences between air and tissue in the brain, which can cause loss of signal and image distortions in certain MR images at these boundaries between tissues.

T1 (T1w), T2 (T2w), and susceptibility *weighted* images can be generated by MR by exciting and acquiring signal at different time intervals. These are sensitive indicators of anatomical structures and T1‐weighted contrast is related to the presence of myelin (Stuber et al., 2014), however, these images are qualitative in nature. Conversely, relaxometry or relaxography refer to quantitative methods that measure and map relaxation times or susceptibility values within tissues, seeking highly accurate and specific measurements of one particular parameter of interest (such as T1 or T2 maps) while removing the confounding effects of other scan parameters and differences in hardware calibration that are present in T1w or T2w images (Alexander et al., 2011).

### Magnetization Transfer Imaging

The MT effect is an observed attenuation of MR signal observed after the application of RF irradiation (Wolff and Balaban, 1989; Grossman et al., 1994). Due to their extremely short T2, many protons in the body, in particular those bound to large macromolecules, are not directly visible in MR images, and the signal from these sources decays away before it can be measured by imaging sequences. However, this pool of macromolecular protons exhibits a much wider range of resonance frequencies, and this property can be exploited to indirectly image them. In MT imaging, an RF pulse is applied either on‐resonance using a wide bandwidth, or more commonly at an offset to the resonance frequency of water, such that it causes a saturation of the protons in the bound pool but not of the free water. To return to equilibrium, this energy imparted to the macromolecules exchanges with free water, resulting in an attenuation in the water signal measured with conventional MR imaging. This effect of exchanging energy that is known as magnetization transfer. Under the assumption that most macromolecular content in the CNS is myelin, the MT effect can thus be exploited as an indirect assessment of myelin content.

### Magnetization Transfer Imaging Ratio (MTR)

The most straightforward MT experiment, known as MT ratio imaging or MTR, is to take an image with a single MT saturation pulse applied and normalize it by an image without MT weighting (holding all other parameters constant) (Fralix et al., 1991). Validation studies have demonstrated correlation between MTR and myelin content (Dousset et al., 1992; Dousset et al., 1995), showing large decreases in MTR in areas of experimentally induced demyelination in animal models, but not areas of oedema. Correlations with histopathology have also shown good agreement between MTR and demyelination and remyelination in histology of rat brains (Deloire‐Grassin et al., 2000). The MTR technique is very straightforward to implement, however comparisons between protocols from different studies is difficult as the amount of MT effect is related to choice of off‐resonance RF power and frequency offset. It is also not necessarily specific to myelin, as other changes in tissue relaxation parameters due to inflammation (Brochet and Dousset, 1999; Gareau et al., 2000), oedema (Cook et al., 2004), and activation of immune response (Blezer et al., 2007) have been found to propagate into MTR maps. By adding a third measurement, it is possible to reduce the influence of T1 relaxation on MTR (Helms et al., 2008), however variability will still exist between studies due differing MT pulse properties.

### Quantitative MT (QMT)

Rather than acquiring a single MT‐weighted image, multiple RF amplitudes and off‐resonance frequencies can be used to sensitize images to different portions of the macromolecular spectrum, in a similar way to how different gradient amplitudes and directions sensitize signal to different amplitudes and directions of water motion in diffusion imaging. These measurements can then be fitted to a model to estimate the relative size of the macromolecular pool of protons to free water (pool size ratio, PSR = bound/free water; bound pool fraction *f* = bound/(bound + free)) (Sled and Pike, 2001; Yarnykh, 2002; Tozer et al., 2003). In addition to this bound pool estimate, the T2 relaxation time of the bound pool (T2b), rate of exchange between pools (*k*), and the relaxation parameters of the free water are modeled. Many measurements are required to robustly fit all of the parameters in these models, leading to long acquisition times. Approaches have been developed to simplify models to reduce the required number of measurements, including assumptions about T2b and *f* to reduce the minimum number of required measurements (Yarnykh, 2012), although such assumptions may not necessarily be valid across subjects of differing age or disease status.

The benefit of quantitative modeling of MT parameters is that such measures should be independent across multiple experiments, scanners, and vendor platforms. Numerical simulations using a four‐pool model found that estimates of bound pool fraction (*f)* accurately track semisolid pool size (myelin), and are insensitive to changes in rate of proton exchange between myelin and nonmyelin compartments (Levesque and Pike, 2009). Strong correlation between both MTR, *f* and optical density of myelin stain (Luxol fast blue) were also observed in a study of fixed and unfixed post mortem brains of MS patients (Schmierer et al., 2007), although in unfixed tissues MTR exhibited slightly higher correlation with myelin content than *f*. Studies of the myelin mutant animals, *shiverer* mouse (Ou et al., 2009) and shaking pup (Samsonov et al., 2012), which lack confounding effects of axonal loss seen in models of MS, indicate that *f* may be more specific to changes myelin than MTR.

### Myelin Water Imaging, Relaxometry

MT imaging sensitizes MR images to myelin through the saturation of macromolecular protons not normally visible in MR sequences. However, there are also water protons associated with myelin that are visible in MR, trapped between the finely spaced lipid bilayers of the sheath. The resolution of MR is such that these fine layers cannot be spatially resolved, however in typical MR imaging the signal coming from water trapped within the myelin contributes to 10–15% of the overall signal in a white matter voxel, and this fractional signal contribution is referred to as myelin water fraction (MWF) (MacKay et al., 1994).

T2 relaxation time is related to the local environment of water, and in general more restricted, dense or viscous tissue environments will give rise to shorter T2. In the context of CNS white matter, compartments of different sizes will give rise to different T2 values, with compartments that are smaller and more geometrically restricted exhibiting shorter T2. In CNS white matter, free water (T2 > 120 ms), extracellular water (T2 ∼ 60–90 ms), and myelin water (T2 ∼ 10–50 ms) all have unique ranges of T2, enabling separation of their relative contribution to overall signal (Stewart et al., 1993; MacKay et al., 1994).

### Multi Echo T2 Mapping

The traditional method for mapping T2 in MRI relies on a technique called multi echo T2 (MET2) mapping (Stewart et al., 1993). In this sequence, signal in a thin slice is excited with an RF pulse, and then measured multiple times as it decays away due to T2 relaxation effects. The observed signal is then fitted to a model of multiple nonexchanging compartments with unique T2 values, with 100 logarithmically spaced T2 values (5–300 ms) typically used in this model (Stewart et al., 1993). The relative signal contribution of components with short T2 (< 50 ms), attributed to water trapped within the myelin layers, are summed and divided by total signal to estimate MWF. Extending this technique to multiple slices is also problematic because exciting signal in multiple slices within a sequence will induce MT effects in neighboring slices, confounding measurements.

The specificity of MWF to myelin has been demonstrated through correlation with the optical density of myelin stain (Luxol fast blue) in formalin‐fixed brains of MS patients (Moore et al., 2000; Laule et al., 2006), as well as in high resolution imaging of rat spinal cord (Kozlowski et al., 2008). A more extensive review of MET2 validation studies can be found in (Laule et al., 2007). In spite of high correlations with myelination, the multi‐exponential model used to estimate MWF does not take into account exchange between compartments, and is only valid under the assumption that the duration of measurements over the signal decay takes place at a timescale much shorter than the rate of exchange. Given a typical measurement is 64 ms to 128 milliseconds in duration, this assumption is likely violated, and studies have demonstrated underestimation of MWF due to exchange effects (Levesque and Pike, 2009; Harkins et al., 2012).

### Steady‐State Multicomponent Relaxometry (mcDESPOT)

Although there have been recent advancements in increasing the efficiency of MET2 techniques (Prasloski et al., 2012), it is difficult to obtain whole brain coverage with multiple slices at reasonable spatial resolution using the MET2 acquisition. This may be acceptable if the goal of an exam is to measure the properties of a known lesion or small set of lesions, however this is limiting for applications investigating changes throughout the brain in populations related to aging, disease, or plasticity. To overcome these time and resolution limitations, a technique called multicomponent driven equilibrium single pulse observation of T1 and T2 (mcDESPOT) was proposed (Deoni et al., 2008) to measure multiple T2 compartments using fast 3D volumetric imaging, enabling full brain imaging with isotropic resolution in a reasonable scan time (∼20 minutes, 2 mm isotropic resolution). The advantages of mcDESPOT are fast speed, volumetric coverage, and relatively high resolution, similar to that of DTI. Instead of modeling the signal from each voxel as a large set of discreet compartments over a wide range of T2 values, mcDESPOT assumes each voxel contains only three sources of signal, each with a single discreet T2 value: exchanging compartments of interaxonal/intraxonal water and myelin water, and a nonexchanging bulk water (e.g., CSF) pool (Deoni et al., 2013). Because of the nature of steady‐state sequences, both T1 and T2 must be modeled for each component.

In spite of its resolution and scan time benefits, the model is very complex and may be poorly posed, thus making it difficult to reliably fit to data. Simulation studies have indicated that the precision of the model (variability of numerical estimates) of may be insufficient to capture meaningful changes in MWF (Lankford and Does, 2013), although implicit regularization in the global optimization method used in fitting may contribute to its robustness (Hurley and Alexander, 2014; Deoni and Kolind, 2015). MWF measures from mcDESPOT have been validated in an animal model with induced demyelination (Wood et al., 2016) and myelin mutant model (Hurley et al., 2010). Both studies found significant decreases in MWF in regions of demyelination and dysmyelination. These results are in disagreement with numerical simulations, which show the statistics of the model may not be able to detect such changes (Lankford and Does, 2013; Hurley and Alexander, 2014), and further investigation is needed to address these discrepancies.

### Susceptibility Weighted Imaging (SWI) and Quantitative Susceptibility Mapping (QSM)

Tissues and materials with differing magnetic susceptibility values will influence local MR signal magnitude (intensity) and phase. The phase of MR signal encodes the precession of spins due to local resonance frequency offset, receiver coil phase, and signal demodulation and digitization (conversion from a high‐frequency analog signal to low‐frequency digital representation of signal). Spins near diamagnetic regions will precess at a slightly lower frequency than the resonance frequency of the main magnetic field, while spins near paramagnetic regions will precess at a slightly higher frequency, leading to different accrual of phase in the regions around tissues of different susceptibility values. The signal in an MR image can be sensitised using a gradient echo image, where signal is excited followed by a long delay to allow signal from spins resonating at slightly different frequencies to dephase, resulting in decay of the signal (T2* weighting), or accrue phase, due to shifts in local magnetic field, resulting in phase offset in images. Susceptibility weighted imaging (SWI) is implemented by combining and filtering magnitude and phase information together to enhance the contrast of susceptibility differences in tissues (de Crespigny et al., 1993; Haacke et al., 2004). These differences are driven by a number of factors, including the microstructural organization of tissues, and in the CNS iron (Langkammer et al., 2012) and myelin in WM are major contributors to susceptibility contrast.

While SWI images are sensitised to relative differences in susceptibility between tissues, quantitative susceptibility mapping (QSM) seeks to quantify the susceptibility shift of tissues (typically expressed in Hertz or parts per million, p.p.m., offset from resonance frequency) (de Rochefort et al., 2008; Shmueli et al., 2009). In QSM, the susceptibility offset within a given image voxel affects the entire image, and thus the image cannot be fitted on a voxelwise basis as in previously described relaxometry techniques. Instead, the problem becomes an inversion or deconvolution of the effect of local susceptibility offsets over the entire image phase. This is complicated by the fact that the magnetic field exists everywhere within the imaged volume, but phase can only be measured in regions where tissues generate a signal, leading to an underdetermined inversion problem that requires regularization and filtering to solve. Other confounding factors include the elimination of background phase, and phase offsets in array receive coils. For more technical details of how this process works, see the following review articles: (Liu et al., 2015; Deistung et al., 2017).

A number of studies have validated the use of susceptibility as a marker for myelin in CNS WM. In a study of the postnatal development healthy mice, WM was found to be paramagnetic relative to GM at birth, become increasingly diamagnetic (decreasing susceptibility) during development. In the same study, diamagnetic shifts in susceptibility were highly correlated with postmortem myelin staining (Argyridis et al., 2014). In a study of *in vivo* development of the human brain, it was found that susceptibility of WM in the brain decreases (becomes more diamagnetic) with brain development development, then increases (becomes more paramagnetic) with aging (Li et al., 2014). In the myelin mutant *shiverer* mouse, it was demonstrated that absence of myelin resulted in a dramatic increase in WM susceptibility (more paramagnetic) as measured with QSM (Liu et al., 2011), while another study in the same model showed that changes in susceptibility of WM were highly correlated with changes in histological myelin stained sections (Lodygensky et al., 2012).

In spite of the strong correlations between QSM and WM myelin, it has been shown in the brain that this assumption of an isotropic, scalar susceptibility value within WM is not valid (Liu, 2010; Li et al., 2012), and values measured using QSM are influenced by the orientation of the WM fibers with respect to the main magnetic field. Susceptibility tensor imaging (STI) (Liu, 2010) is a method to quantify anisotropic susceptibility values, similar to how DTI is implemented to quantify the anisotropic diffusion of water in the CNS. STI requires multiple images be acquired with the sample placed at different orientations with respect to the main magnetic field of the magnet. Because, in most magnet designs, the direction of the main field is fixed along the direction of the magnet bore, this requires the subject or sample be reoriented in the scanner, something that is difficult to achieve for human subjects during in‐vivo scanning, greatly limiting the number and orientation of directions that can be acquired. Finally, it is known that iron deposition occurs in multiple sclerosis and Alzheimer's disease (LeVine, 1997), and using QSM or STI for quantification of changes in myelin may be confounded in the presence of these pathologies.

### Myelin Mapping Methods

The previous methods described have relied on sensitizing the MR acquisition to specific relaxation properties of tissue, either by indirect measurement of the short T2 macromolecular‐bound proton pool, or separation of short T2 signals from myelin water. An alternative approach to relaxometry for mapping myelin in the CNS is to use the ratio of standard structural images to produce maps of myelination. A map can be derived from the MR signal intensity of a T1‐weighted (T1w) image normalized (divided) by a T2‐weighted (T2w) image registered into the same space to remove bias due to effects such as the receiver coil and enhance contrast, and it has been found that spatial distribution of signal intensities from this map co‐localise with myelin‐stained histology from the cortical surface (Glasser and Van Essen, 2011). This method of myelin mapping was shown to delineate boundaries of cortical areas in population‐average maps as well as individual subjects, albeit with less precision. The presence of myelin has been shown to reduce both T1 and T2 times in peripheral nerves (Jolesz et al., 1987), however both parameters are also related to overall water content in tissues, and thus T1w and T2w images individually have low specificity to myelin. Quantitative maps of R1 (1/T1) have also been shown to demonstrate high co‐localisation with myelinated regions (Lutti et al., 2014) are in theory less sensitive to variations in choice of scan parameters and scanner hardware. Ratio images for mapping cortical myelin may however exhibit increased sensitivity to myelin through combinations of multiple types of myelin contrast present in the imaging sequences utilized. Iron is co‐localised with myelin in the cortex (Fukunaga et al., 2010), contributing to T2w image contrast, and MT effects are also present in the T2w CUBE/SPACE acquisition typically utilized for myelin mapping techniques (Thomas et al., 2004; Weigel et al., 2010). A detailed review of studies investigating correlations of biological features with cortical myelin mapping can be found in (Glasser et al., 2014).

The benefit of this approach is that the structural images typically acquired in a routine MR exam (i.e., for segmentation, registration, and screening of incidental findings) can also be used to estimate myelin content. It is also possible to get very high resolution images (∼1 mm cubic) in a reasonable scan time, which is not compatible with previously mentioned methods that require many measurements to fit a model. This may be important for looking at cortical myelination, rather than WM, where higher resolution is necessary to accurately delineate the folded cortical surface.

### Comparison of Techniques

Several of the most popular techniques for quantitatively estimating myelin content in the CNS have been discussed, falling broadly into the three categories of magnetization transfer imaging, myelin water imaging, susceptibility mapping, and cortical myelin mapping (Table 1). Myelin itself is not directly MR visible, and MT, MWI, and QSM techniques measure myelination indirectly, through protons bound to macromolecules in MT, water trapped within the lipid bilayers of the myelin sheath in MWI, and shifts in tissue susceptibility values in QSM. In contrast, myelin mapping generates relative maps of cortical myelination through the combined contrast of multiple nonquantitative structural imaging sequences. Quantitative (QMT and MWF) techniques rely on complex modeling of the MR signal dependence on myelin, and require more measurements and thus more scan time than simple ratio techniques such as MTR or myelin mapping. However, quantitative modeling, if performed properly, should remove confounding effects of scan parameters and hardware variability.

Although MT and MWI are based on different phenomena, both show high correlation with myelin content in the CNS, and numerical simulations (Levesque and Pike, 2009) show that both methods are sensitive to changes in CNS myelination. In vivo imaging in MS patients and healthy controls has also revealed high correspondence between bound pool fraction from QMT and MWF from MET2 imaging (Tozer et al., 2005), although between‐subject variability was found to be lower for QMT due to more robust fitting of the model. Additionally, MWF is sensitive to not only changes in the relative size of the myelin water compartment, but also exchange between compartments, while QMT is shown to be less sensitive to exchange effects (Dula et al., 2010). Simulations show that MWF estimates decrease linearly with increasing exchange, while QMT measures are not affected (Levesque and Pike, 2009). It is important to keep in mind that both MT and MWF rely on estimates of the relative fraction of bound or myelin water protons to overall water content, and changes in the bulk water content of tissues will influence estimates from either set of techniques.

Methods based on MT and MWI are primarily designed to evaluate changes in WM myelination, and most validations have been performed in demyelinating diseases such as MS or animal models of demyelination and dysmyelination. In contrast, the technique of myelin mapping through signal ratios was developed to evaluate patterns of cortical myelination, and validated against the spatial distribution of histological measures of myeloarchitecture (Geyer et al., 2011). It is thus important to keep in mind that these two techniques have been developed with different goals in mind.

In summary, magnetization transfer imaging and QMT offer the potential for highly sensitive and specific, although indirect, measures of myelin in the CNS, and steady‐state QMT methods offer whole‐brain coverage with moderate spatial resolution. However, due to the complexity of MT models, many acquisitions are required, leading to long acquisition times and requiring customized pulse sequences. MWI methods are also sensitive to myelin, although unlike QMT, MET2 models are underdetermined and require regularization to solve. Traditional spin‐echo MWI techniques are limited to one or several thick slices, making it difficult to easily achieve whole‐brain coverage, and require long acquisition times (e.g., 20+ minutes per slice). Steady‐state MWI methods (i.e., mcDESPOT) enable rapid, whole‐brain coverage of myelin water mapping (e.g., 15–20 minutes for 2 mm isotropic whole‐brain coverage), but may suffer further from unstable fitting, and is in need of further validation. Both MT and MWI methods require the implementation of custom MRI pulse sequences. In contrast, SWI, QSM, and myelin mapping methods rely on standard sequences provided by most MR vendors. In QSM, a single multi‐echo three dimensional acquisition can easily achieve whole‐brain coverage at moderate to high spatial resolution however, like MWI, is also an underdetermined inversion problem requiring careful choice of regularization. Finally, myelin mapping techniques rely on standard structural MRI images, and thus offer the highest spatial resolution, shortest scan times, and most straightforward protocol implementation. However, these methods are based on validations through correlation of cortical myelin features, and may not be as quantitative as the aforementioned methods.

## MEASURING G‐RATIO WITH MRI METHODS

The myelin g‐ratio is the ratio of the radius of the axon to the radius of the axon plus the myelin sheath (Rushton, 1951). The g‐ratio of an axon affects its ability to conduct electrical signals with an optimal value in the CNS estimated to be around 0.77 (Chomiak and Hu, 2009). The MRI g‐ratio is a simplified aggregated measure of the myelin g‐ratio based on the assumption that all the myelinated axons in a voxel are uniform (Stikov et al., 2015). A voxel of white matter can be divided into the myelin volume fraction (MVF), the part that contains the myelin, the axon volume fraction (AVF), the part occupied by the axons, and the fiber volume fraction (FVF), containing both the axons and their myelin sheaths. The MVF and AVF or the FVF can be used to calculate the MRI g‐ratio (Stikov et al., 2015). A method to determine the MRI g‐ratio using quantitative magnetization transfer (qMR) to estimate the MVF and neurite and orientation dispersion and density imaging (NODDI) to determine the AVF has been developed (Stikov et al., 2015). Using this method, the MRI g‐ratios in a healthy human brain and that of a macaque monkey were found to be around 0.7 fitting with the estimated optimal value (Chomiak and Hu, 2009), whereas new MS lesions were found to have g‐ratios above 0.8 (Stikov et al., 2015). Comparisons of ex‐vivo histological and MRI g‐ratios in the macaque corpus callosum were found to be very similar, although the MRI g‐ratio was marginally higher overall (Stikov et al., 2015).

An alternative method for estimating the MRI g‐ratio using quantitative multi‐parameter mapping (MPM) of magnetization transfer data to find the MVF and tensor fiber density (TFD) of diffusion weighted images to determine the FVF has been developed (Mohammadi et al., 2015). This was used to map a number of white matter tracts in healthy participants. The corticospinal tract, superior longitudinal fasciculus and fornix were found to have MRI g‐ratios of 0.65 or higher, whereas the cingulum, inferior occipitofrontal fasciculus and optic radiation had MRI g‐ratios that were less than 0.65. The pattern of variation in MRI g‐ratios differed from the pattern for magnetization transfer (MT), TFD and fractional anisotropy (FA) measures obtained for the same tracts, demonstrating that the MRI g‐ratio can contribute additional information about white matter structure (Mohammadi et al., 2015). The corpus callosum was divided into eight regions of interest (ROI) with the MRI g‐ratios found to compare fairly well with ex‐vivo findings in the macaque monkey (Stikov et al., 2015). The pattern of variation in g‐ratio across different ROIs within the corpus callosum was also similar to the pattern for TFD and FA maps, whereas MT values showed a different pattern (Mohammadi et al., 2015). Another modified method for estimating the MRI g‐ratio using multicomponent driven equilibrium single pulse observation of T1 and T2 (mcDESPOT) to determine the MWF and NODDI for the FVF has also been developed (Dean et al., 2016).

## MYELIN MR IMAGING APPLICATIONS

### Imaging Myelin Development in Humans

A number of studies have made use of advanced imaging methods to assess myelin during different stages of human development. Note that there have been many studies conducted using DTI to assess changes over the lifespan (e.g., (Barrick et al., 2010; Giorgio et al., 2010; Qiu et al., 2010; Sullivan et al., 2010; Sexton et al., 2014) and these have been extensively reviewed elsewhere (Carmichael and Lockhart, 2012; Dubois et al., 2014; Moseley, 2002; Qiu et al., 2015). We will focus our discussion in this and future sections on neuroimaging studies that have employed the more myelin‐specific MR measures described above. The majority of these studies have been cross‐sectional, so myelin differences are inferred, and therefore longitudinal studies are needed. However, progress is being made in assessing myelination over the human lifespan with the links between myelination and cognitive ability also being explored.

The relationship between age and myelin during very early development and childhood has been investigated. Myelin mapping using T1w/T2w ratio was calculated in a number of cortical regions in new born babies between the postconceptual ages of 40–48 weeks (Lee et al., 2015). Increases in myelin were found with age in the cerebellar, temporal, occipital and parietal cortices but not in the corpus callosum or cingulum (Lee et al., 2015). MRI g‐ratios (aggregated across each voxel) throughout the developing brain have also been calculated in healthy children aged from 3 months to 7 years (Dean et al., 2016). Overall, the MRI g‐ratio was shown to decrease (indicating increased myelination) rapidly in the first 15 months and then decrease more slowly between the ages of 15 months to 7 years (Dean et al., 2016). The MRI g‐ratio was found to decrease in different brain regions at different rates. For example, more internal brain structures such as the internal capsule and corpus callosum decreased quicker from 3 months compared with cortical regions (Dean et al., 2016). This is in keeping with previous histological findings showing that brain myelination develops in a centre‐out pattern (Kinney et al., 1988). In another study, the MWF was obtained using multi component relaxometry in a cross‐sectional and longitudinal design to compare cognitive ability and white matter myelination during early development (Deoni et al., 2016). The MWF and general cognitive ability were found to be correlated in different brain regions at different ages. For example, in toddlers (12–24 months) increased myelin in the corticospinal tract, primary motor, somatosensory, visual, auditory and cerebellar regions was correlated with increased cognitive ability, whereas in young children (2–5 years) the somatosensory, right pre‐motor cortex and anterior cingulum were correlated with cognitive ability (Deoni et al., 2016). The MWF in splenium of the corpus callosum was found to be correlated with cognitive ability across all ages studied (Deoni et al., 2016). Myelin development was slower initially in the above average cognitive ability children throughout the first year of life, but later increased with these children having greater MWF compared to children assessed to be average or below average ability (Deoni et al., 2016).

Intra‐cortical myelin during different stages of life has been examined (Grydeland et al., 2013). T1w/T2w along with DTI derived mean diffusivity measures were obtained for participants between the ages of 8 and 83. Increase in T1w/T2w ratio values, indicative of myelin maturation, was found to continue into the late thirties, and stabilize between the ages of 30 and 50 before gradually declining in later years (Grydeland et al., 2013). This inverted U shape trend of myelin maturation was also found in other studies using T1 or T2 relaxometry (Bartzokis et al., 2012; Yeatman et al., 2014). R1 (1/T1) was found to increase between the ages of 7 and 40, peak between the ages of 30 and 50, before declining. This decline was found to mirror the increases seen during development with 80 year olds having similar R1 values to 8 year olds (Yeatman et al., 2014). R2 (1/T2) along with the DTI derived FA, radial, axial and mean diffusivity were obtained for three ROI. The frontal white matter and the genu of the corpus callosum, areas that have been shown by postmortem histology to be myelinate at later ages and have higher myelin content, along with the earlier myelinated splenium of the corpus callosum, which has lower myelin content (Bartzokis et al., 2012). R2 was the only measure found to demonstrate this pattern of myelin content in the three ROI (Bartzokis et al., 2012). In another study, multi‐shell diffusion weighted MRI and QMT were used to quantify the MRI g‐ratio in different brain regions including the anterior thalamic radiation, corticospinal tract, inferior fronto‐occipital fasciculus, superior longitudinal fasciculus and forceps (Cercignani et al., 2017). The MRI g‐ratio was found to increase with age in these tracts with the 20 to 40 year olds having a lower MRI g‐ratio compared with middle‐aged participants (41–60 years) and those over 60 (Cercignani et al., 2017). Age‐related changes in myelin measures have also been shown to relate to cognitive performance. For example, in adults aged over 52, reduced myelin (indicated by a lower T1/T2 ratio) was correlated with increased intra‐individual variability in reaction time over trials (Grydeland et al., 2013).

### Imaging Experience‐Dependent Myelin Plasticity in Humans

Changes in white matter with skill acquisition have been widely reported in neuroimaging studies in humans (Scholz et al., 2009; Taubert et al., 2010) with rodent studies suggesting that myelin might be involved in the observed changes (Sampaio‐Baptista et al., 2013). Recently, advanced imaging methods are starting to be used to assess changes in myelin following either skilled or general physical exercise as well as working memory training. In one study, the MWF was determined using multicomponent T2 relaxation imaging before and after extensive skilled motor training using the right arm over a four‐week period (Lakhani et al., 2016). The MWF in the left intraparietal sulcus (IPS) and parieto‐occipital sulcus (POS) increased after training (Lakhani et al., 2016), suggesting an experience‐dependent increase in myelination in these areas.

A relation between myelin changes and physical exercise has remained less clear so far. A few cross sectional studies suggest a correlation between white matter structure (assessed using DTI) and amount of physical activity in older adults (Burzynska et al., 2014; Tian et al., 2015), though as with any cross‐sectional study it is hard to assess the specificity of this relationship. Very few studies have yet used more myelin‐specific MR measures to assess relationships between physical activity and white matter microstructure. A recent, cross‐sectional study in young adults found a positive correlation between MWF in the right parahippocampal cingulum and amount of physical activity monitored over three days (Bracht et al., 2016). There is need for more interventional studies in humans to assess whether such relationships reflect a causal influence of physical activity on myelination. Still, in the rodent literature it has been reported that running promotes OPCs proliferation and differentiation in gray and white matter regions (Ehninger et al., 2011; Matsumoto et al., 2011; Simon et al., 2011; McKenzie et al., 2014), suggesting a causal association between aerobic exercise and myelination which should be further explored in humans.

The effects of working memory training over a period of eight weeks on white matter has also been investigated (Caeyenberghs et al., 2016). A number of structural brain networks were generated using a variety of imaging methods including diffusion MRI and mcDESPOT. Participants were split into two training groups, a high capacity group in which task difficulty increased based on performance and a nonadaptive group in which task difficulty was independent of performance (Caeyenberghs et al., 2016). Adaptive training was found to improve complex working memory and verbal reasoning and increase global network connectivity. The anterior cingulate gyrus and right inferior ventrolateral prefrontal cortex were found to be more locally connected (Caeyenberghs et al., 2016). This change in the brain networks was only picked up by the mcDESPOT derived R1 and R2 measures and was thus suggested to be more specific to changes in oligodendrocytes as T2 weighted images pick up iron content and oligodendrocytes have a higher level of iron compared to other cells (Caeyenberghs et al., 2016). In a further study by the same group, adaptive working memory training led to increased R1 and diffusion weighted contrasts including FA but not MWF in the superior longitudinal fasciculus and parahippocampal cingulum (Metzler‐Baddeley et al., 2017).

### Imaging Myelin in Neuropathology and Psychiatric Disorders

Myelin imaging has a potentially important role in the clinic. Demyelinating disorders including MS are a common cause of disability and noninvasive methods to monitor disease progression or predict response to therapy are needed (Filippi et al., 2013a, 2013b). In addition, altered myelin development or degeneration may play an underappreciated role in a number of developmental or degenerative neuropsychiatric disorders (Haroutunian et al., 2014).

MT has been used extensively to study multiple sclerosis (MS) (For reviews see (Ropele and Fazekas, 2009; Mallik et al., 2014; Gass et al., 2015). MTR, a proposed marker of myelin and axonal loss, has been shown to correlate with MS disability (Gass et al., 1994). However, the MTR can potentially be affected by oedema and or inflammation (Vavasour et al., 2011). Two alternative measures of myelin have been described using QMT the fraction of macromolecular protons (f_B_,) and relaxation (T2) of the macromolecular pool (T_2B_) (Tozer et al., 2003). Differences in both the f_B_ and T_2B_ were shown between MS lesioned white matter and controls (Davies et al., 2004). Differences in f_B_ were also found in normal appearing white matter between controls and MS patients in the frontal, occipital, corpus callosum and internal capsule, whereas differences in T_2B_ were found in the occipital cortex (Davies et al., 2004). Myelin content in unfixed postmortem brain slices from patients with MS have been used to validate MTR measures. The MTR and f_B_ were found to correlate well with the transmittance of Luxol‐fast blue, a stain for myelin (Schmierer et al., 2004; Schmierer et al., 2007). Conversely, T_2B_ was not found to correlate (Schmierer et al., 2007). The MTR and f_B_ were compared in postmortem brain slices either fixed with formalin or left unfixed (Schmierer et al., 2010, 2008). The MTR in both gray and white matter was found to be higher in nonfixed compared to fixed tissue, whereas f_B_ was found to be lower. Caution should therefore be taken when comparing in‐vivo results with formalin fixed ex‐vivo histology (Schmierer et al., 2010). In another study, MWF and volume of the brain and spinal cord of primary progressive MS patients and healthy controls were obtained using mcDESPOT (Kolind et al., 2015). Changes in brain volume (suggested to indicate atrophy) were found to correlate with cognitive processing, evaluated using Paced Auditory Serial Addition Test and dexterity, evaluated using the 9‐Hole Peg Test (9‐HPT). MWF (indicating myelin) was found to correlate with 9‐HPT performance in the corpus callosum (Kolind et al., 2015). The MWF and f_B_ however, have been found to only partially correlate, indicating that one or both measures may not specifically measure myelin but could also be affected by other factors such as axonal loss or inflammation (Tozer et al., 2005).

Advanced MRI methods have also been used to assess other neurodegenerative pathologies. Cerebral MWF and T2 intra/extra (I/E T2) cellular water measures were obtained using mcDESPOT in patients with primary lateral sclerosis (PLS) and amyotrophic lateral sclerosis (ALS) (Kolind et al., 2013). MWF was found to be lower, suggestive of reduced myelin, in PLS patients compared with those with ALS and healthy controls. Compared with controls ALS had increased I/E T2 water, suggestive of inflammation (Kolind et al., 2013). These two measures could therefore potentially be used as biomarkers to distinguish between PLS and ALS (Kolind et al., 2013). In another study, the MWF, R1 and R2 were obtained using mcDESPOT in participants with Parkinson's disease (Dean et al., 2016). Increases in these measures were found in a number of regions including the corpus callosum, frontal cortex, internal capsule and thalamus. However, it was acknowledged that these changes could be influenced by the participants' use of medication (levodopa) as myelin changes have been linked to dopaminergic activity (Dean et al., 2016). MTR has also been used to assess the contralesional hemisphere in stroke patients (Lin et al., 2015). Changes in the MTR of motor connections were found to occur within a month, indicating myelin degeneration secondary to the stroke lesion (Lin et al., 2015). Chronic stroke patients were found to have reduced MWF in total cerebrum white matter as well as the posterior limb of the internal capsule (Borich et al., 2013).

Studies investigating psychiatric disorders have also employed advanced MRI myelin imaging methods with mixed results. Schizophrenia patients were found to have higher MTR in the inferior frontal‐occipital fasciculus, right uncinate fasciculus and arcuate fasciculus in participants with schizophrenia (Mandl et al., 2015). However, no differences were found in the free water content (water located in the extracellular space) or FA making it difficult to conclude whether the changes in MTR were due to free water or myelin (Mandl et al., 2015). Furthermore, increased T1 relaxation in the globus pallidus but not frontal white matter has been demonstrated in participants with schizophrenia (Spaniel et al., 2005). However, small participant numbers were used and the authors acknowledged that the patients' use of antipsychotic medication could have altered activity in the globus pallidus, likely accounting for the changes observed (Spaniel et al., 2005). Another study found no difference in the MWF in the frontal white matter during the early stages of schizophrenia (Lang et al., 2014).

MTR has also been assessed in patients with later life major depressive disorder (MDD). A lower MTR was found in a number of brain regions including the prefrontal and occipital white matter, cingulate regions, insular, thalamus and corpus callosum of older depressed patients (Kumar et al., 2004; Gunning‐Dixon et al., 2008).

## CONCLUSIONS

In the last few decades we have seen a rapid development in MRI techniques that allow for more specific quantification of myelin in the living brain. Continuing technical advances will offer higher specificity and sensitivity, faster acquisitions and higher resolution. As such, validation is a critical step in establishing new techniques for measuring myelin to determine specificity and sensitivity, for instance, through histological myelin quantifications in both human and animal models, in healthy and pathological tissue. Studies in healthy tissue correlating myelin content with imaging measures are useful for assessing specificity to myelin, as well as studies in demyelinating diseases such as multiple sclerosis (MS). Animal models are also critical to understanding the biological effects that drive changes in MR measures, and enable longitudinal imaging. The administration of a neurotoxin to induce CNS demyelination is a common model for the validation of myelin imaging techniques, and cuprizone is a common toxin used to achieve this (Skripuletz et al., 2011). This is particularly useful for precise control over the onset of demyelination and subsequent remyelination in longitudinal studies, although such models may have limited utility in assessing specificity due to confounding effects such as inflammation, oedema, and axonal loss. These issues of specificity can in part be overcome by studying models with genetic mutations that lack myelin during development (dysmyelination) without confounding effects, such as the *shiverer* mouse (Bird et al., 1978; Rosenbluth, 1980), *taiep* rat (Duncan et al., 1992), and *shaking* pup (Bray et al., 1983). Further, numerical simulations are useful for validating mathematical models of MR signal, evaluating the performance of model fitting in the presence of measurement noise, and determining optimal imaging parameters for a given technique. Although, MRI will never achieve the same resolution and specificity of cellular techniques, such as immunohistochemistry and microscopy, the ability to measure myelin in the human brain *in vivo* offers huge opportunities for the understanding of brain development, ageing, plasticity and pathology and for monitoring the efficacy of interventions and therapeutics.

This work was supported by Wellcome Principal Research Fellowship to HJB (WT110027/Z/15/Z).
